# A compact quad-element serrated boundary fractal planar antenna for multi-band mmWave 5G/6G wireless applications

**DOI:** 10.1371/journal.pone.0349601

**Published:** 2026-06-03

**Authors:** Tathababu Addepalli, Thota Vidyavathi, Maragani SatishKumar, Gudapati Divya, Morasa Balaji, Sivasubramanyam Medasani, Mohammad Sami Zidan, Imran Mohd Ibrahim, Ahmed Jamal Abdullah Al-Gburi

**Affiliations:** 1 Department of ECE, Aditya University, Surampalem, India; 2 Department of ECE, Anil Neerukonda Institute of Technology and Sciences (A), Sanghivalasa, Visakhapatnam, Andhra Pradesh, India; 3 Department of ECE, SRKR Engineering College, Bhimavaram, Andhra Pradesh, India; 4 Department of ECE, Bapatla Women’s Engineering College, Bapatla, Andhra Pradesh, India; 5 Department of ECE, School of Engineering, Mohan Babu University [Erstwhile Sree Vidyanikethan Engineering College], Tirupati, Andhra Pradesh, India; 6 Department of CSE, K. S. School of Engineering and Management, Bengaluru, India; 7 Department of Electrical Techniques, Technical Institute of Anbar, Middle Technical University, Baghdad, Iraq; 8 Center for Telecommunication Research & Innovation (CeTRI), Fakulti Teknologi Dan Kejuruteraan Elektronik Dan Komputer (FTKEK), Universiti Teknikal Malaysia Melaka (UTeM), Jalan Hang Tuah Jaya, Durian Tunggal, Melaka, Malaysia; 9 Strategic Research Institute (SRI), Asia Pacific University (APU), Jalan Teknologi 5, Taman Teknologi Malaysia, Kuala Lumpur, Malaysia; 10 Faculty of Informatics Engineering, Syrian Private University (SPU), Damascus, Syria; Galgotias College of Engineering and Technology, Greater Noida, INDIA

## Abstract

This paper proposes a compact serrated boundary fractal planar quad-element MIMO antenna engineered for multi-band millimeter-wave (mmWave) 5G/6G systems. The structure is designed and developed on 30 × 30 mm^2^ size rogers’ material of thickness 0.8 mm. The proposed design evolves progressively through three stages, from a conventional rectangular patch to a compact, fractal-inspired geometry featuring embedded slots and symmetrical serrated arrow-shaped protrusions. This structural evolution significantly enhances electromagnetic coupling, current path diversity, and multi-band resonance behaviour. The antenna resonates at four distinct mmWave frequency bands 24.5 GHz, 33.5 GHz, 38.0 GHz, and 44.0 GHz, covering key portions of the 5G spectrum. The compact quad-element layout exhibits high isolation, notable peak gain, and favourable diversity metrics, including ECC (Sim ≤ 0.00008, Mea ≤ 0.00010), DG (Sim ≤ 10 dB, Mea ≤ 10 dB), TARC (Sim ≤ −10 dB, Mea ≤ −9 dB), CCL (Sim ≤ 0.005 bits/s/Hz, Mea ≤ 0.010 bits/s/Hz), and MEG (Sim ≤ −3 dB, Mea ≤ −3 dB), all within ITU-recommended limits, collectively contributing to robust MIMO performance. Its compact size, structural symmetry, and multiband performance make it an excellent candidate for low-latency, and interference-resilient wireless applications in next-generation vehicular and IoT communication systems.

## 1 Introduction

Millimeter-wave (mmWave) technology has gained widespread attention for its potential to provide high bandwidth, low latency, making it well-suited for ultra-fast data transmission and sensing applications [1,2]. Its versatility enables deployment across a wide spectrum of communication and non-communication sectors. The 5G New Radio (NR) standard incorporates several millimeter-wave (mmWave) frequency bands, including n257 (26.5–29.5 GHz), n258 (24.25–27.5 GHz), n259 (39.5–43.5 GHz), n260 (37.0–40.0 GHz), and n261 (27.5–28.35 GHz), which are specifically established for high-speed, low-latency 5G mmWave applications [3,4]. Fractal antennas [5–9] have emerged as a promising solution for next-generation wireless systems, utilizing sharp, self-similar notches inspired by natural fractal patterns to achieve targeted resonance at multiple frequencies, making them ideal for 5G and beyond. The geometries most frequently employed in the design of fractal antennas are star [10,11], Sierpinski Gasket/Carpet, a triangular based structure [12,13], Giuseppe Peano antenna based on Peano curves [14,15], Minkowski island, a modified square structure [16,17], Koch curve, a snowflake-like pattern [18,19], Hilbert curve, a space-filling curve [20,21], Cantor set, line segment-based fractal [22,23]. Each fractal geometry holds its own importance in designing fractal antennas for specific wireless applications. However, a single fractal geometry alone cannot achieve multiband or wideband characteristics without affecting antenna performance. To address this, researchers have combined multiple fractal geometries to create unique antenna designs tailored for various wireless applications.

Sierpinski fractal-based Microwave Metamaterial Absorber (MMA) [24] that demonstrates dual-band operation, specifically at X and K bands resonating at 8.2GHz and 20.24 GHz. A new shape of Koch–Sierpinski fractal mmWave antenna [25] is suggested to operate effectively within the frequency range of 27.55 GHz to 28.6 GHz, with a resonant frequency at 28 GHz, and demonstrates excellent mutual coupling characteristics, maintaining values below −25 dB. A CPW-fed fractal UWB antenna [26] for biomedical applications operates efficiently from 3.2 to 20 GHz through wedged slots in the radiating patch. A new design of microstrip patch antenna operated on multi band frequencies, using the star hexagon fractal concept is proposed by Subramanian *et al*. [27] operating in the frequency range from 24.9 GHz to 28.1 GHz with a peak gain of 4.688 dB at 28 GHz is presented. Mallat [28] introduced a unique Fractal Arrow-Shaped mmWave Antenna based on Flexible material for IoT and 5G systems operating at a wideband in the range of 15 GHz to 40 GHz. A compact auxiliary Koch fractal dipole wideband antenna [29] using coaxial-to-parallel-strip transition is developed to be used for the frequency range 2.3–6 GHz in order to cover various wireless bands. And some other works, which are related to fractal antennas are presented [30–42]. A single monopole antenna of size 22 × 26 mm^2^ is designed for super wide band application using Sierpinski Triangular Fractal method [43]. A triple band antenna is designed for sub 6 GHz, WIFI and WiMAX application is presented [44]. Design and development CPW fed of a Modified Hilbert Curve, 50 × 60 × 3 mm^3^ size Fractal Antenna for Multiband Applications is presented [45]. Owing to their compact size, multiband capability, and high isolation, fractal antennas are exceptionally well-suited for millimeter-wave 5G and emerging 6G applications, where space efficiency and precise frequency tuning are essential.

The current research outlines a compact serrated-boundary fractal patch antenna, which incorporates primary and secondary discrete serrations along the radiating structure in synergy with a peripherally notched octagonal parasitic disc at the center. The multi-level serration technique effectively perturbs surface currents, while the central parasitic disc couples with the surrounding radiators to suppress mutual coupling, broaden impedance bandwidth, and stabilize radiation patterns. Together, these attitudes enable multiband operation with enhanced radiation efficiency, isolation, and overall antenna performance. The suggested MIMO antenna resonates at 24.5 GHz, 33.5 GHz, 38.0 GHz, and 44.0 GHz, effectively covering multiple mm-Wave bands relevant to 5G and beyond. It obtains high port-to-port isolation exceeding 20 dB and obtains notable gain values between 8.1 dBi and 9.8 dBi, enabling robust radiation performance with minimal mutual coupling in a MIMO system. The integration of discrete serrated boundaries with fractal configuration further improves bandwidth, efficiency, and isolation, making the design a good option for next-generation wireless systems, including 5G/6G communications, V2X connectivity, and IoT applications.

## 2 MIMO antenna design and analysis

**Fig 1:**
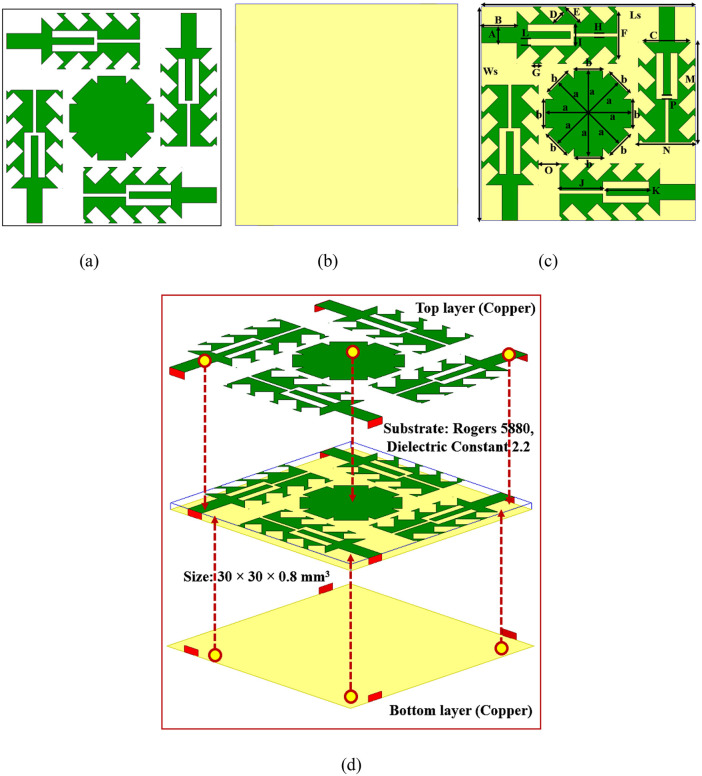
The configuration MIMO antenna, (a) front layer, (b) back layer, (c) with parameters and (d) 3D-Dimetric view.

The modelled Quad-port Serrated Boundary Fractal Patch (SBFP) MIMO antenna, developed for high-performance working in the 5G/6G mm-Wave bands, is presented in Fig 1. The novelty of this design lies in its fractal patch geometry with discrete serrated boundaries, incorporating both primary and secondary serrations to enhance multiband performance. A tuning-fork-shaped slot is modelled at the center of the SBFPA to enhance impedance matching and suppress mutual coupling. Furthermore, the orthogonal arrangement of the four SBFP elements is centered around a peripherally-notched octagonal parasitic disc, which contributes to bandwidth broadening, improved isolation, and stabilized radiation patterns. The top and bottom layers of the design are introduced in Fig 1(a) and 1(b), while the detailed dimensions and 3D isometric view are illustrated in Fig 1(c) and 1(d). The antenna is developed on a Rogers RT5880 laminate with a laminate height of 1.575 mm, and a relative permittivity $\epsilon_{r}=\text{2}.\text{2}$, with a loss tangent tan δ = 0.0009, and is excited using a 50-Ω microstrip feed line for proper impedance matching. The key design parameters are recorded in Table 1, and the description for all geometries is seen in Table 2. With compact overall sizes of 30 × 30 × 0.8 mm^3^, the antenna is highly suitable for next-generation mm-Wave systems.

**Table 1:** Geometrical parameters of the suggested MIMO antenna (Units: mm).

Variables	A	B	C	D	E
**Magnitude**	*2.2*	*5*	*4.2*	*2*	*3*
**Variables**	**F**	**G**	**H**	**I**	**J**
**Magnitude**	*6.4*	*0.6*	*0.5*	*3*	*6*
**Variables**	**K**	**L**	**M**	**N**	**O**
**Magnitude**	*6.5*	*1*	*14*	*8*	*3*
**Variables**	**P**	**L**_**S**_	**W**_**S**_	**a**	**b**
**Magnitude**	*0.5*	*30*	*30*	*12*	*4*

**Table 2:** Description of all MIMO antenna design.

Variables	Specification	Magnitude (mm)
**A**	Width of the Feed Line	2.2
**B**	Length of the Feed Line	5
**C**	Width of the Radiating Patch	4.2
**D**	Width of the Outer Serration	2
**E**	Depth of Outer Serration	3
**F**	Width of the Radiator up to Primary Serrations	6.4
**G**	Width of the Secondary Serrations	0.6
**H**	Width of the Stem of Tuning Fork Slot	0.5
**I**	Length of the Yoke of Tuning Fork Slot	3
**J**	Length of the Stem of Tuning Fork Slot	6
**K**	Length of the Prongs of Tuning Fork Slot	6.5
**L**	Width of the Prongs of Tuning Fork Slot	1
**M**	Length of the Radiator	14
**N**	Width of the Radiator	8
**O**	Spacing between the Radiators	3
**P**	Width of the Yoke of Tuning Fork Slot	0.5
**L**_**S**_	Length of the Substrate	30
**W**_**S**_	Width of the Substrate	30
**a**	Length of Rectangular Patch	12
**b**	Width of Rectangular Patch	4
**g**	Spacing between the prongs	

**Fig 2:**
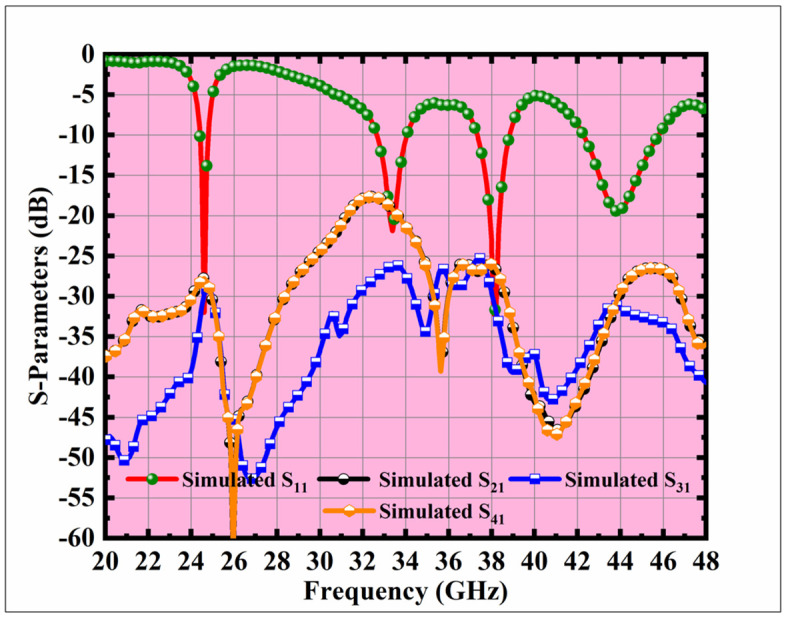
MIMO antenna S-parameter simulation results.

The proposed antenna resonates at 24.5 GHz (n258: 24.25–27.5 GHz), 33.5 GHz (emerging 6G exploratory band), 38.0 GHz (n260: 37–40 GHz), and 44.0 GHz (candidate 6G band), thereby covering key portions of the 5G spectrum while extending into future 6G allocations. The simulated S-parameters, as shown in Fig 2, confirm excellent impedance matching with S_11_ values of –42 dB, –32 dB, –39 dB, and –22 dB at the respective resonant frequencies. In addition, the design demonstrates strong isolation performance with inter-port isolation levels of 48 dB at 24.5 GHz, 35 dB at 33.5 GHz, 36 dB at 38.0 GHz, and 28 dB at 44.0 GHz, consistently exceeding the 20 dB benchmark. These results highlight the antenna’s ability to provide multiband operation, low mutual coupling, and robust MIMO performance.

### 2.1 MIMO antenna evolution process

**Fig 3:**
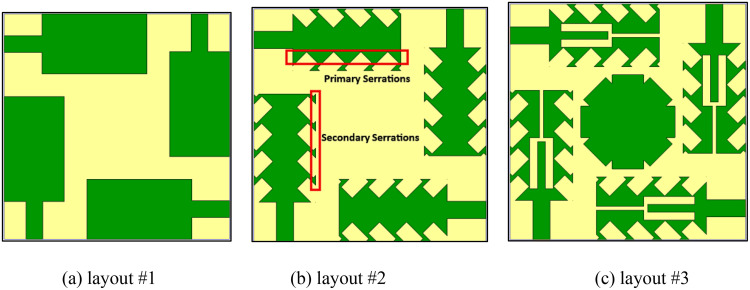
MIMO antenna parametric stages.

The architecture of the modelled quad-port antenna Serrated Boundary Fractal Patch (SBFP) MIMO antenna is carried out through three stages, as shown in Fig 3, with each stage focused on enhancing key performance metrics such as return loss, impedance bandwidth, isolation and gain. The introduction of discrete primary and secondary serrations along with a peripherally notched octagonal parasitic disc progressively improves the antenna’s multiband response and overall radiation performance.

Fig 3(a) presents Antenna #1, a four-port orthogonally oriented rectangular patch antenna designed using the standard transmission-line model equations for microstrip patches [30]. This configuration is developed as the fundamental structure for the proposed design.

Width of the radiating patch is computed using


$$\begin{array}{c} W=\frac{c}{\text{2}f_{r}}\sqrt{\frac{\text{2}}{\epsilon_{r}+\text{1}}} \end{array}$$ (1)

Where,

c = velocity of light

$\epsilon_{r}$ = relative permittivity of the substrate

$f_{r}$ = resonant frequency of the antenna

Length of the radiating patch is calculated by using the following equation


$$\begin{array}{c} L=L_{eff}-\text{2}\Delta L \end{array}$$ (2)

Where,

$L_{eff}$ = effective length of the antenna

ΔL = extended length of the antenna

The effective length of the antenna at the specified frequency is evaluated using the following equation


$$\begin{array}{c} L_{eff}=\frac{c}{\text{2}f_{r}\sqrt{\epsilon_{eff}}} \end{array}$$ (3)

Where,

$\epsilon_{eff}$ = effective dielectric constant of the antenna, quantified using


$$\begin{array}{c} \epsilon_{eff}=\frac{\epsilon_{r}+\text{1}}{\text{2}}+\frac{\epsilon_{r}-\text{1}}{\text{2}}\left(\frac{\text{1}}{\sqrt{\text{1}+\frac{\text{2}h}{W}}}\right) \end{array}$$ (4)

The extended length of the radiating rectangular patch is determined by using the equation


$$\begin{array}{c} \Delta L=h*\text{0}.\text{4}\text{1}\text{2}*\frac{\left(\epsilon_{eff}+\text{0}.\text{3}\right)\left(\frac{W}{h}+\text{0}.\text{2}\text{6}\text{4}\right)}{\left(\epsilon_{eff}-\text{0}.\text{2}\text{5}\text{8}\right)\left(\frac{W}{h}+\text{0}.\text{8}\right)} \end{array}$$ (5)

Antenna #1 exhibits five distinct resonances at 21.35 GHz, 24.70 GHz, 31.93 GHz, 35.96 GHz, and 43.95 GHz, with return loss values ranging from –11.80 dB to –20.85 dB. The associated impedance bandwidths fall between 0.52 GHz and 2.85 GHz, and inter-element isolation remains better than 24 dB across all operating bands. Despite its multiband behaviour, the achieved resonances are not fully aligned with the intended 5G and emerging 6G mmWave spectrum allocations, indicating the need for additional design refinement.

As shown in Fig 3(b), Antenna #2 employs a two-stage serration strategy. In the first stage, inward triangular serrations are patterned along the radiator edges to form the primary modification. In the second stage, additional outward notches are introduced at the tips of the primary serrations, creating the secondary serration profile. This multi-level serrated structure results in three distinct resonances at 32.82 GHz, 39.45 GHz, and 44.73 GHz, with return loss values between –13.15 dB and –18.00 dB. Bandwidths increase up to 3.47 GHz, and isolation remains above 25 dB. Although the design shifts the resonances toward higher frequencies and improves bandwidth, it does not generate additional lower-band resonances required for comprehensive coverage. Antenna #3, shown in Fig 3(c), integrates a tuning-fork-shaped slot etched at the center of the four radiators and a peripherally notched octagonal parasitic disc at the center of the configuration. This modification enables stable quad-band operation at 24.5 GHz, 33.5 GHz, 38.0 GHz, and 44.0 GHz. The achieved return loss values are significantly improved (–19.49 dB to –32.25 dB), with bandwidths ranging from 0.39 GHz to 3.50 GHz. Importantly, isolation performance is also enhanced, remaining above 26 dB across all four bands. These results confirm that Antenna #3 provides superior impedance matching and isolation, ensuring reliable operation in both 5G mmWave and transitional 6G frequency ranges.

**Fig 4:**
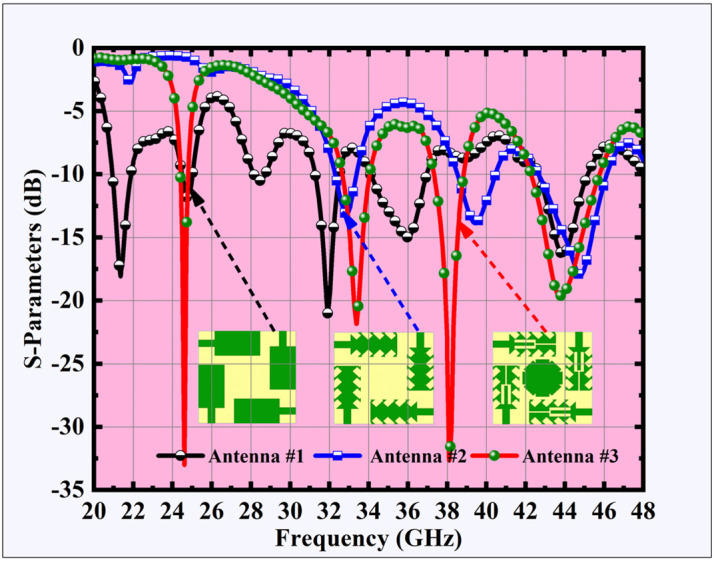
S-parameter of MIMO antenna design stages.

The resonant frequencies of the three antenna configurations, along with their corresponding return loss values and bandwidths and high isolation levels are recorded in Table 3 and listed in Fig 4.

**Table 3:** Performance comparison of Antenna #1, 2 & 3.

Antenna	${𝐟}_{r}$ (GHz)	${𝐒}_{11}$ (dB)	Bandwidth (GHz)	Isolation (dB) (S_31_)
**Antenna # 1**	21.35	−18.07	0.92	37.40
	24.70	−11.80	0.52	24.15
	31.93	−20.85	1.23	24.82
	35.96	−14.97	2.85	27.71
	43.95	−16.37	2.50	30.31
**Antenna # 2**	32.82	−13.15	1.07	25.20
	39.45	−13.86	1.92	26.35
	44.73	−18.00	3.47	28.29
**Antenna # 3**	24.5	−32.01	0.39	29.80
	33.5	−21.88	1.40	26.25
	38.0	−32.25	1.50	30.80
	44.0	−19.49	3.50	31.15

### 2.2 Influence of geometrical parameters on MIMO antenna performance

To further estimate the performance of the suggested Quad-port SBFP MIMO antenna, a parametric study is carried out by varying key geometrical parameters. The study mainly focuses on the influence of serration depth, serration width, the tuning-fork slot length, and the dimensions of the central peripherally notched octagonal disc on the antenna’s characteristics. These parameters directly affect the resonant frequencies, impedance bandwidth, isolation levels, and radiation efficiency. The analysis provides critical insights into design sensitivity, ensuring optimized performance across the targeted 5G and transitional 6G mmWave bands.

#### 2.2.1 Effect of width of the feed (A).

**Fig 5:**
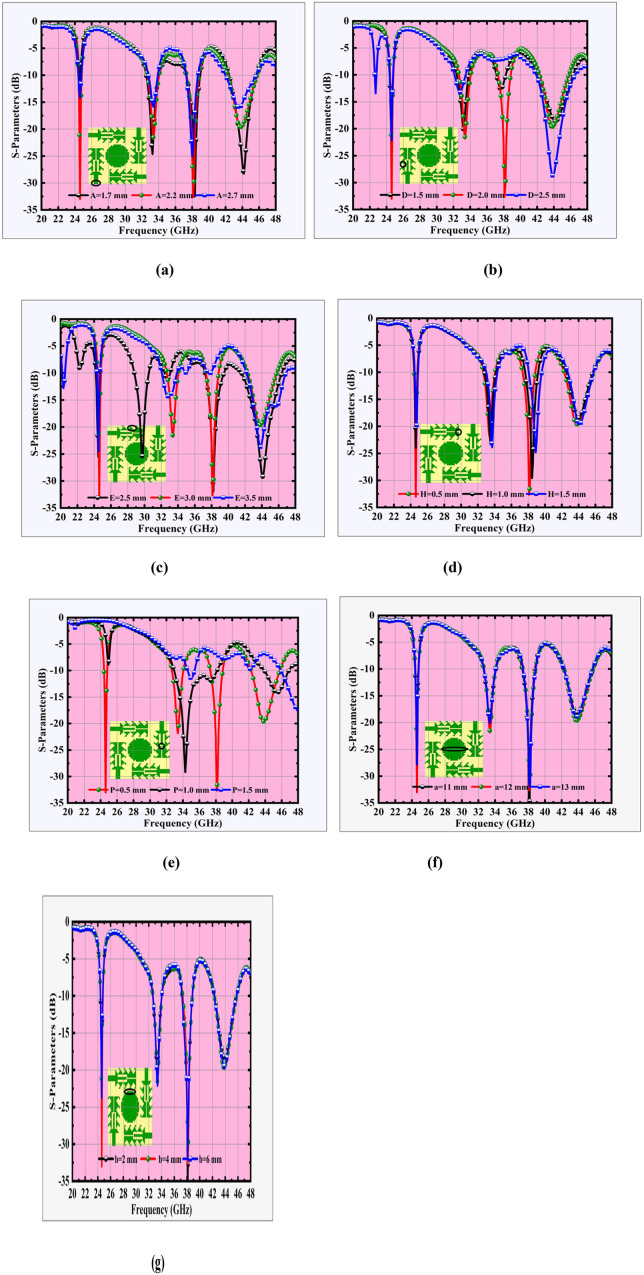
(a)-(f) MIMO antenna parametric analysis.

The width of the microstrip feed line plays a vital role in determining the characteristic impedance and ensuring proper impedance matching with the antenna. Variations in feed width directly influence the return loss, resonant frequencies, and overall bandwidth performance. Fig 5(a) offers the simulated return loss characteristics when the feed width (A) is varied as 1.7 mm, 2.2 mm, and 2.7 mm. It is observed that the optimal feed width of 2.2 mm provides superior impedance matching, with deeper return loss levels across the resonant bands. A narrower feed width of 1.7 mm results in impedance mismatch at higher bands, leading to performance degradation, while a wider feed width of 2.7 mm shifts the resonances and weakens matching, particularly around 38–44 GHz. Hence, proper feed width selection is essential for achieving 50 Ω matching, stable multiband operation, and enhanced bandwidth in the designated 5G and transitional 6G mmWave bands. An optimal feed width of 2.2 mm ensures a balanced trade-off between impedance matching and bandwidth, thereby making it the most suitable choice for the proposed antenna design.

#### 2.2.2 Effect of outer serration width (D).

The outer serration width (D) significantly influences the impedance characteristics and resonance behavior of the suggested antenna. Fig 5(b) illustrates the predicated return loss response for three various values of D = 1.5 mm, 2.0 mm, and 2.5 mm. It is observed that the optimal serration width of D = 2.0 mm provides deeper return loss values and better impedance matching across the three resonant bands, especially at 24.6 GHz and 38 GHz, thereby ensuring stable multiband operation. A narrower serration width of 1.5 mm results in a frequency shift and poor impedance matching at the higher band, indicating inadequate coupling due to reduced slot interaction. Conversely, a wider serration width of 2.5 mm causes over-coupling, leading to distorted impedance characteristics and degraded return loss performance, particularly around 32–36 GHz.

#### 2.2.3 Effect of outer serration depth (E).

The outer serration depth (E) strongly influences the antenna’s impedance matching by altering the balance between edge capacitance and path inductance. Increased edge area enhances fringing fields and raises capacitance, while deeper serrations force longer, irregular current paths that increase inductance. With a minimal serration (E = 2.5 mm), weaker fringing fields and shorter current paths yield lower capacitance and inductance, favouring higher-frequency resonance and improved matching in the 44–46 GHz band. In contrast, a pronounced serration (E = 3.5 mm) introduces excessive discontinuities, elevating inductance and disturbing the capacitance–inductance balance, which degrades performance across most bands. The optimized case, E = 3.0 mm as depicted in Fig 5(c), provides the right balance delivering strong impedance matching across all key operating frequencies.

#### 2.2.4 Effect of stem width of tuning fork slot (H).

The stem width H of the tuning fork slot controls the coupling between the prongs and the radiator. As shown in Fig 5(d), the optimal width of H = 0.5 mm achieves the deepest return loss and strong impedance matching at the operating bands 24.5 GHz, 33.5 GHz, 38 GHz, and 44 GHz. Increasing H to 1.0 mm and 1.5 mm weakens the resonances, particularly at 33.5 GHz and 38 GHz, due to over-coupling and resonance shifts. Hence, H = 0.5 mm is the most suitable choice for stable multiband performance and enhanced bandwidth in the 5G and transitional 6G mmWave bands.

#### 2.2.5 Effect of yoke width of tuning fork slot (P).

The yoke width (P) is a critical coupling parameter, which is fundamentally important in tuning the resonances of the antenna because it directly controls both the effective current path length and the slot capacitance at the junction (yoke) of the tuning fork prongs. The tuning fork slot can be modelled as a parallel LC resonator, with the resonant frequency given by


$$\begin{array}{c} f_{r}\approx \frac{1}{2\pi \sqrt{L_{eff}C_{eff}}} \end{array}$$ (6)

Where,

$L_{eff}$ is the effective inductance associated with the current path around the slot

$C_{eff}$ is the capacitance across the gap.

The capacitance is approximately proportional to


$$\begin{array}{c} C\;\alpha \frac{\in_{eff}L}{g} \end{array}$$ (7)

Where,

L is the slot width and

g is the spacing between the prongs

At smaller values of P, the electric fields are highly confined at the yoke, which increases the effective capacitance $C_{eff}$ and lengthens the current path $L_{eff}$, lowering the resonances beyond the desired bands. Conversely, larger values of P reduce both capacitance and effective length, shifting the resonances upward and weakening the impedance match. At P = 0.5 mm, the balance between capacitance and inductive current path satisfies the LC condition such that the resonances align precisely with the target bands at 24.5, 33.5, 38, and 44 GHz, giving deeper return loss and stable performance as illustrated in Fig 5(e).

#### 2.2.6 Effect of length and width of central parasitic patch (a & b).

The central parasitic patch is constructed by superimposing a rectangular patch of length *a* and width *b*, rotated at angles 0°, 45°, 90°, and 135° with respect to the reference axis. This arrangement produces an peripherally notched octagonal-shaped parasitic disc with discrete serrated-like edges. The overlapping geometry introduces multiple current paths and controlled edge perturbations, which enhance the effective electrical length of the radiator and improve bandwidth. The parameters *a* (octagon side length) and *b* (serration width) thus serve as critical tuning variables for the resonant behaviour of the antenna.

As shown in Fig 5(f), increasing a enlarges the current path, lowering the resonant frequencies. For a = 11 mm, the resonances shift upward, while a = 13 mm lowers the resonances excessively, degrading return loss. The optimum a = 12 mm provides strong impedance matching with resonances aligned at 24.5 GHz, 33.5 GHz, 38 GHz, and 44 GHz. Fig 5(g) shows the effect of serration width b. A smaller b = 2 mm produces insufficient perturbation, leading to shallow return loss at higher bands, while a larger b = 6 mm causes excessive perturbation, distorting resonances around 33–38 GHz. The optimum b = 4 mm achieves the best balance, enhancing bandwidth and maintaining stable return loss across all bands. Thus, the combination of a = 12 mm and b = 4 mm ensures that the central octagonal parasitic patch resonates efficiently, enabling stable multiband performance and deeper return loss in the desired 5G and transitional 6G mmWave spectrum.

### 2.3 MIMO antenna SCD response

**Fig 6:**
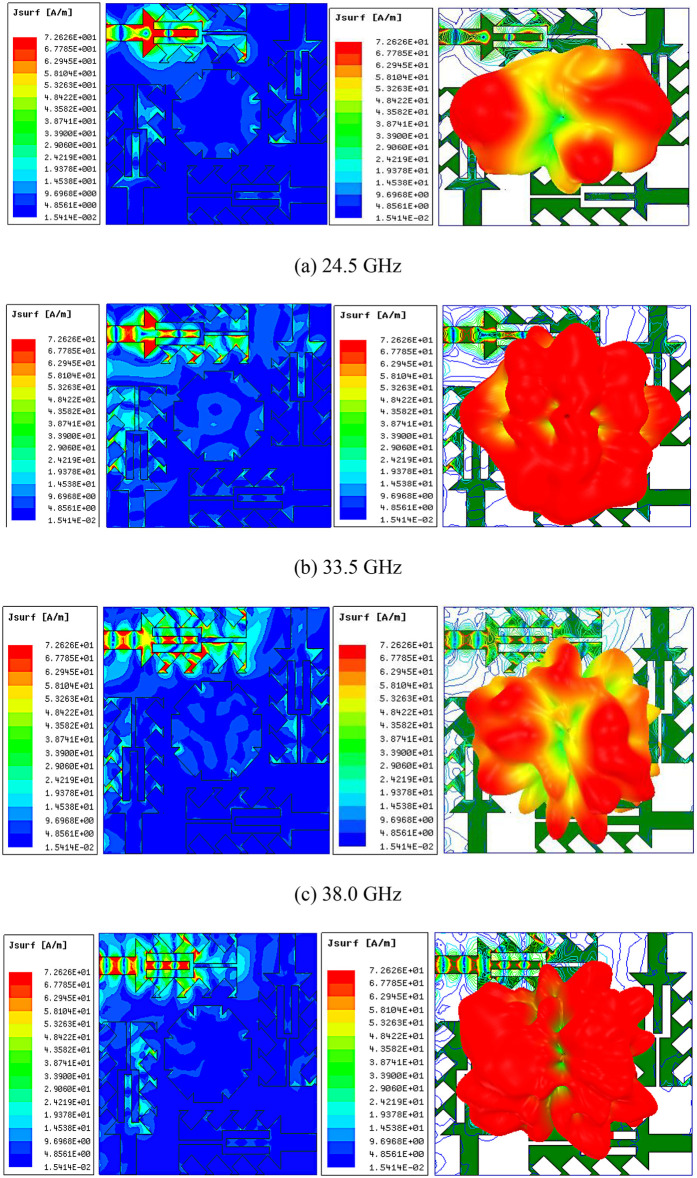
(a)-(d) MIMO antenna surface current distribution analysis.

Fig 6 provides the 2D and 3D surface current distributions of the suggested MIMO antenna at its four resonant frequencies. In Fig 6(a), at 24.5 GHz, strong current density is concentrated around the feed line, tuning fork yoke, indicating that these regions dominate the excitation of the fundamental resonance. The currents remain largely confined to the radiator. which directly corresponds to the high isolation of 29.8 dB observed at this frequency. This validates that the antenna geometry effectively suppresses coupling in the lower band by restricting the excitation primarily to the driven element.

As represented in Fig 6(b), at 33.5 GHz, the currents are highly concentrated around the feed line, tuning fork slot and tips of the secondary serrations and adjacent radiator. This mutual interaction results in an isolation of 26.15 dB at the observed resonance.

As portrayed in Fig 6(c), at 38 GHz, strong currents appear along the feed line, tuning fork slot, spacing between the prongs, and the tips of both primary and secondary serrations, while the central disc carries relatively weaker currents. Regions of maximum current density near the prongs and serrations extend partly toward the adjacent element. The high isolation of 30.8 dB at 38 GHz is achieved because the antenna geometry effectively suppresses continuous coupling paths between the radiators.

Fig 6(d) shows the surface current distribution of the antenna at 44 GHz. Strong current concentration is observed along the feed line, tuning fork slot, and the spacing between the prongs, while the central disc and non-excited regions carry comparatively weaker currents. The serration tips also support noticeable currents. A portion of current slightly couples toward the adjacent radiator. However, due to the optimized slot and serration geometry, strong current transfer is restricted. As a result, the antenna obtains a high isolation of 31.15 dB, ensuring effective suppression of mutual coupling at this frequency.

### 2.4 Effect of octagonal disc/stub on gain and impedance matching

**Fig 7:**
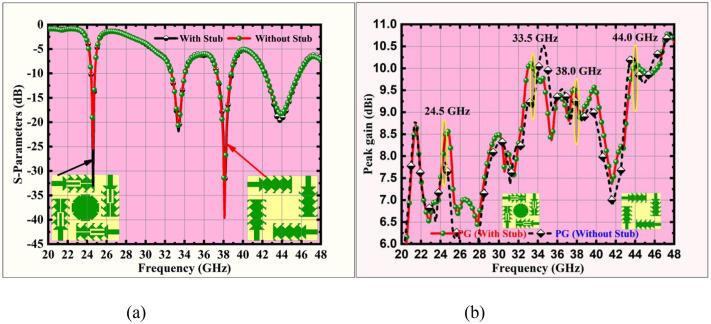
(a)-(b) MIMO antenna octagonal disc effect response.

Fig 7(a) illustrates the return loss characteristics without and with tuning fork slot and the peripherally notched octagonal parasitic disc. The antenna with these features exhibits improved impedance matching and reduced reflection losses. The corresponding peak gain performance is presented in Fig 7(b), where antenna attains gains of 8.45 dBi at 24.5 GHz, 10.01 dBi at 33.5 GHz, 9.23 dBi at 38.0 GHz, and 10.07 dBi at 44.0 GHz. These results clearly demonstrate that the incorporation of the tuning fork slot and parasitic disc enhances both impedance matching and radiation efficiency through the operating bands.

Peak gain values without and with inclusion of peripherally notched octagonal parasitic disc is tabulated in Table 4. At 24.5 GHz, the gain improves from 7.85 dBi to 8.45 dBi, showing that the central disc helps enhance radiation efficiency in the lower band. At 33.5 GHz, a notable improvement is also observed, with the gain increasing from 9.13 dBi to 10.01 dBi, highlighting the disc’s effectiveness in supporting resonance at this band. At 38 GHz, the gain remains unchanged at 9.23 dBi, indicating that the central disc has minimal influence on this frequency. Similarly, at 44 GHz, the gain shows a negligible change (10.15 dBi without disc and 10.07 dBi with disc), suggesting that higher-band performance is largely unaffected. Overall, the central disc contributes to improved gain in the lower and mid-bands, while maintaining stable performance in the higher bands.

**Table 4:** Comparison of peak gain values of the antenna at resonant frequencies with and without the central disc.

Resonant Frequency (GHz)	Peak Gain without Central Disc	Peak Gain with Central Disc
**24.5**	7.85	8.45
**33.5**	9.13	10.01
**38**	9.23	9.23
**44**	10.15	10.07

## 3. Results and discussions

### 3.1 Near field response

**Fig 8:**
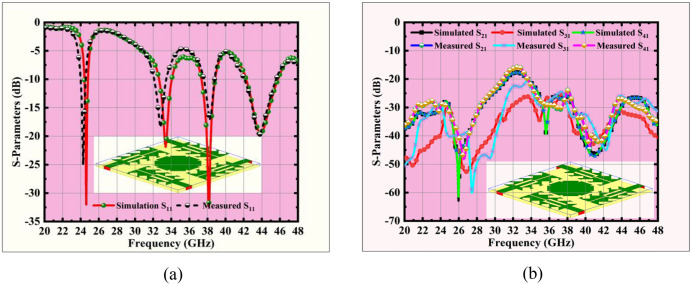
MIMO antenna performance comparison (a) S_11_ Comparison. IMO, and **(b)** Isolation Comparison.

The predicted and tested S-parameter characteristics of the finalized MIMO antenna are presented in Fig 8(a), showing close agreement between results. The antenna exhibits multiple resonant bands at approximately 24.5 GHz, 33.5 GHz, 38 GHz, and 44 GHz, with return loss values well below –10 dB, confirming efficient impedance matching and quad-band operation. Additionally, the isolation performance, shown in Fig 8(b), S_21_, S_31_, and S_41_ remains consistently better than 17 dB, demonstrating good suppression of mutual coupling among ports. These results validate the antenna’s design for reliable high-performance operation in 5G and emerging 6G applications.

The predicted and tested isolation properties of the finalized MIMO antenna show good correlation at all resonant frequencies, demonstrating the reliability of the design as recorded in Table 5. The values remain closely aligned, with only slight variations observed. These small mismatches arise mainly due to fabrication tolerances, connector losses, substrate property variations, and measurement setup limitations. Despite these differences, both simulated and measured results consistently maintain isolation well above 18 dB across all operating bands, confirming effective suppression of mutual coupling and ensuring stable MIMO performance.

**Table 5:** Simulated & measured isolation levels.

Frequency (GHz)	S_21_ (dB)		S_31_ (dB)		S_41_ (dB)	
	Simulated	Measured	Simulated	Measured	Simulated	Measured
**24.5**	27.74	28.47	29.80	30.48	27.77	28.59
**33.5**	19.02	18.93	26.23	25.74	19.03	18.06
**38**	26.36	25.20	30.80	32.11	26.25	25.47
**44**	30.60	28.80	31.15	29.00	30.49	29.88

**Fig 9:**
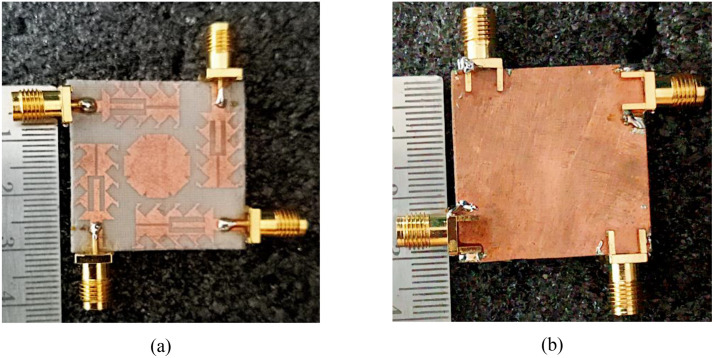
(a) Front prototype, and (b) Back prototype of the finalized fractal MIMO antenna.

The prototype of the proposed Quad-port Serrated Boundary Fractal Patch (SBFP) MIMO antenna is presented in Fig 9. Fig 9(a) depicts the top view and Fig 9(b) illustrates back view of the finalized antenna.

### 3.2 Far field response

**Fig 10:**
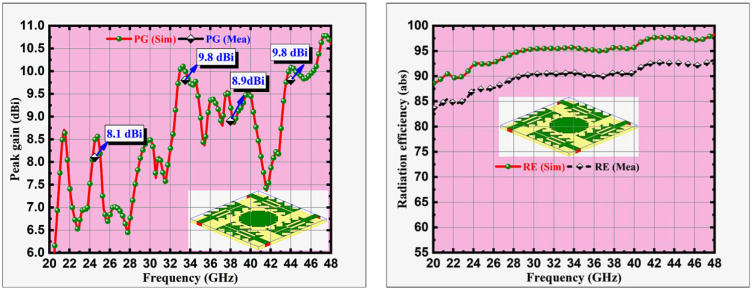
Sim & Meas MIMO antenna (a) Peak gain (dBi), and (b) Radiation efficiency (abs).

The predicted and tested peak gain values and radiation efficiencies at the corresponding frequencies are represented in Figs 10(a), 10(b) respectively. The peak gain values of 8.1 dBi, 9.8 dBi, 8.9 dBi and 9.8 dBi and simulated radiation efficiency values of 92.5%, 95.6%, 95.5% and 97.6% are observed at corresponding resonant frequencies 24.5, 33.5, 38 and 44 GHz.

**Fig 11:**
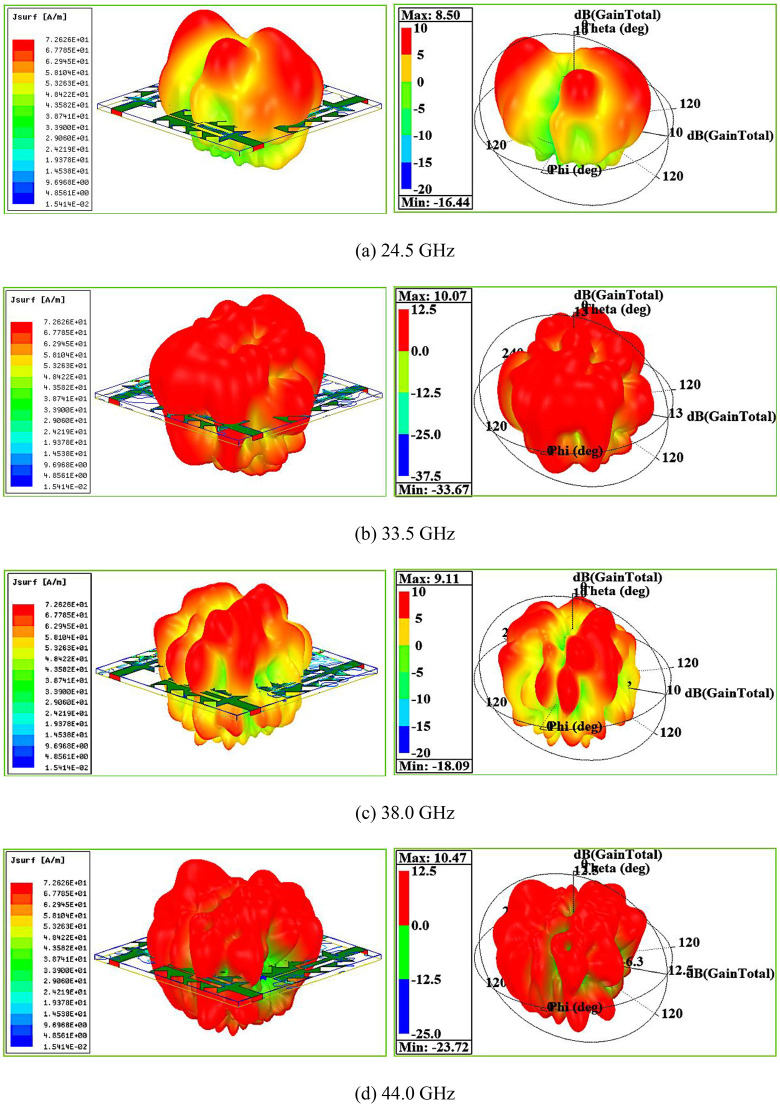
(a)-(d) MIMO antenna 3D-polar plot analysis.

The MIMO antenna 3D polar plots at the resonant frequencies 24.5 GHz, 33.5 GHz, 38 GHz and 44 GHz are displayed in Fig 11(a)-11(d).

**Fig 12:**
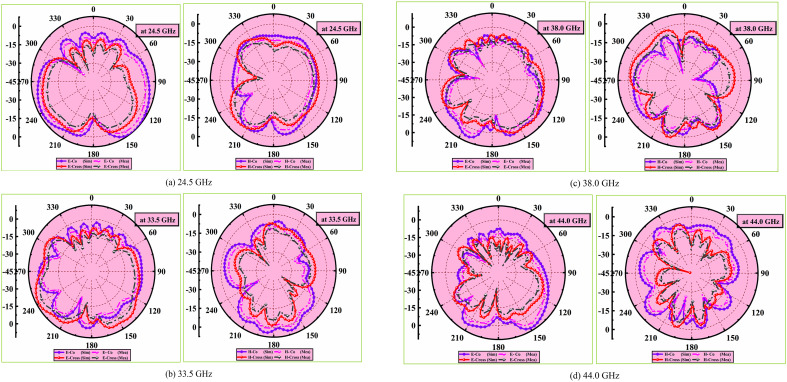
(a)-(d) MIMO antenna 2D-radiation pattern plot analysis.

**Fig 13:**
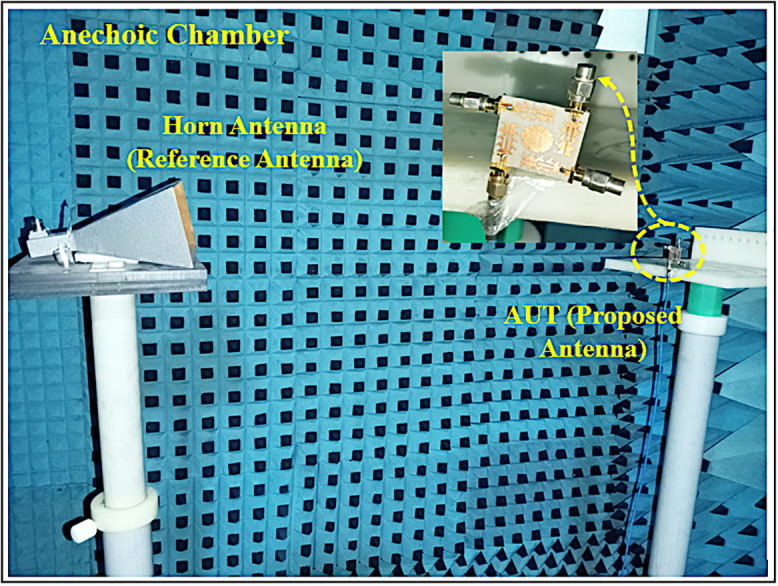
Fabricated MIMO antenna in a chamber for testing.

The predicted and tested 2D radiation plots in E and H planes including co-polarization and cross-polarization components at four resonant frequencies 24.5 GHz, 33.5 GHz, 38 GHz and 44 GHz are exhibited in Fig 12(a)-12(d). The finalized MIMO antenna in the chamber for testing is shown in Fig 13.

The performance comparison of the suggested Quad-port SBFP MIMO antenna with the current published antennas in the literature [31–34,40–42] is outlined in Table 6. The proposed serrated boundary fractal antenna with inclusion of tuning fork shaped slot and peripherally notched octagonal parasitic disc is the novel structure of the antenna. The orthogonal orientation of the elements in a compact sized provides good radiation characteristics in the 5G mm-Wave band and transitional 6G future bands

**Table 6:** Comparison with relate works.

Ref	Band	No. of Elements	Substrate with$\varepsilon_{r}$	Resonant Frequencies (GHz)	Fractal Structure	Isolation (dB)	Gain (dBi)
[28]	Tri-Band	1	Cotton $\epsilon_{r}=\text{1}.\text{6}$	16.5, 24.5, 36.5	Arrow	–	4.5
[31]	Single	1	FR4 $\epsilon_{r}=\text{4}.\text{4}$	27	Minkowski	–	5.8
[32]	Dual-Band	4	FR4 $\epsilon_{r}=\text{4}.\text{4}$	29.81, 34.29	Fractal Loaded	30	6.4, 5.3
[33]	Single	4	–	23.56–28.25	–	21	6.65
[34]	Tri-Band	4	Rogers $\epsilon_{r}=\text{2}.\text{2}$	28, 37, 48	Minkowski	<−10 for all bands	6.9, 7.5, 6
[40]	Multi-Band	2	FR4 $\epsilon_{r}=\text{4}.\text{4}$	9.5, 11.1, 13.4, 15.8, 21.1, 26.6	Tweaked Spherical	<−16 for all bands	Max gain 8.46
[41]	Multi-Band	4	FR4 $\epsilon_{r}=\text{4}.\text{4}$	22.4, 34.5, 42.4, 48.8, 50.6	Cross Fractal	<−17 for all bands	Max gain 5.72
[42]	Quad-Band	4	FR4 $\epsilon_{r}=\text{4}.\text{4}$	2.85, 4.75, 6.3, 7.81	Koch Fractal	<−15 for all bands	1.7, 2.6, 4.1, 3.9
**Proposed**	**Quad-Band**	**4**	**Rogers** $\epsilon_{r}=2.2$	**24.5, 33.5, 38, 44**	**Serrated Boundary**	**29.8, 26.2, 30.8, 31.1**	**8.1, 9.8, 8.9, 9.8**

### 3.3 MIMO Metrics

**Fig 14:**
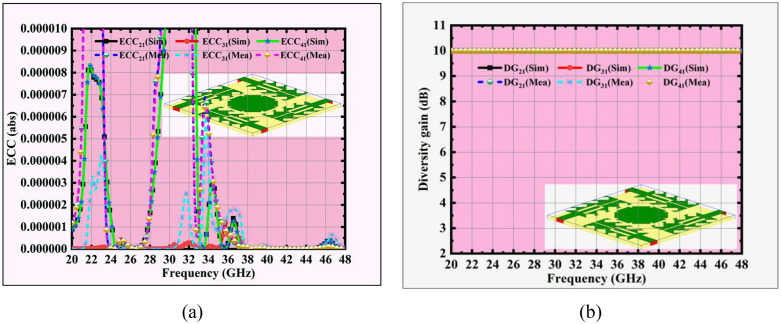
Sim and meas MIMO antenna (a) ECC, and (b) DG responses.

**Fig 15:**
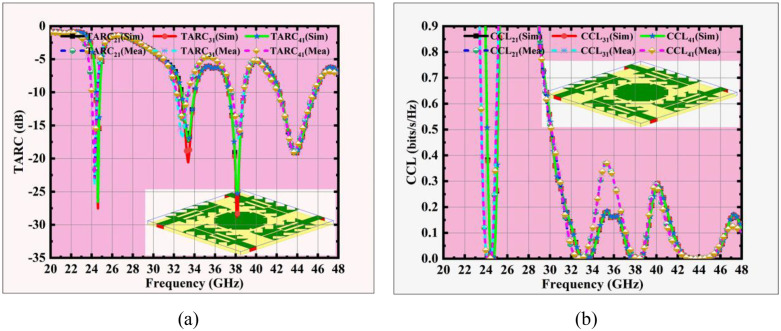
Sim and Meas MIMO Antenna (a) TARC, and (b) CCL responses.

**Fig 16:**
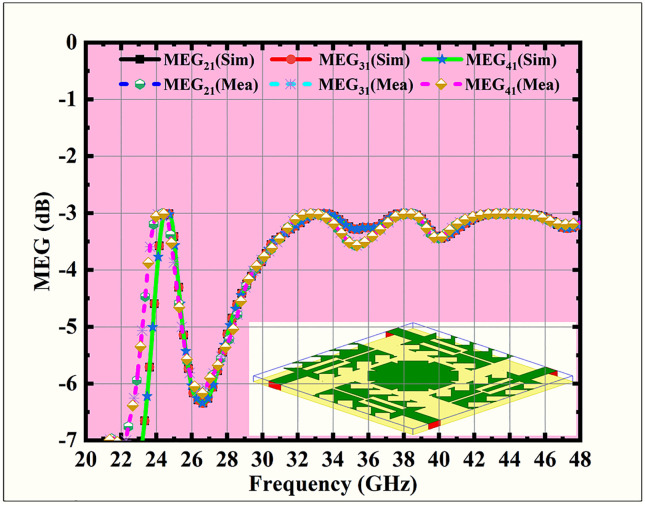
MIMO antenna MEG response.


The predicted and tested MIMO performance metrics are presented in Figs 14–16. Among these, the Envelope Correlation Coefficient (ECC) is one of the key parameters that quantifies the degree of correlation between adjacent antenna patches. The ECC in terms of S-parameters [35] is calculated using:

$$\begin{array}{c} \rho_{e}=\frac{{\left|{S_{\text{1}\text{1}}}^{*}S_{\text{1}\text{2}}+{S_{\text{1}\text{2}}}^{*}S_{\text{2}\text{2}}\right|}^{\text{2}}}{\left(\text{1}-{\left|S_{\text{1}\text{1}}\right|}^{\text{2}}-{\left|S_{\text{2}\text{1}}\right|}^{\text{2}}\right)(\text{1}-{\left|S_{\text{2}\text{2}}\right|}^{\text{2}}-{\left|S_{\text{1}\text{2}}\right|}^{\text{2}})} \end{array}$$ (8)

where, S_11_ is the reflection coefficient and

S_12_ is the isolation

A lower ECC magnitude provides better isolation and diversity performance between MIMO antenna ports, ensuring that the antenna elements operate more independently, which is essential for reliable MIMO operation. For a practical MIMO antenna system, the ECC threshold should be ≤ 0.5, while for advanced 5G and 6G applications, it is ideally expected to be **<** 0.1, ensuring better isolation and superior diversity performance. From Fig 14(a), it is evident that simulated ECC values of 0.0000010, 0.00000085, 0.00000065 and 0.00000050 are observed at 24.5 GHz, 33.5 GHz, 38 GHz and 44 GHz.

Diversity Gain (DG) [36] is another important MIMO performance metric that indicates how effectively multiple antenna elements improve the overall signal reliability. It is mathematically related to the ECC and is expressed as


$$\begin{array}{c} DG=\text{1}\text{0}\sqrt{\text{1}-|\rho {|}^{\text{2}}} \end{array}$$ (9)

Ideally, DG values remain close to 10 dB, and when ECC is very low, the DG approaches this maximum value, confirming superior diversity performance and reliable operation for MIMO systems. Fig 14(b) reveals a consistent DG value of 10 dB across the operating bands, further validating the antenna’s capability to deliver excellent diversity and robust MIMO performance.

TARC is another MIMO metric, which represents the reflection behaviour of a MIMO antenna system when all ports are excited with equal amplitude but different phases. It accounts for both reflection and coupling, making it an important metric to evaluate multi-port antenna performance. In terms of incident and reflected waves TARC [37] is given by the formula


$$\begin{array}{c} TARC=\sqrt{\frac{\sum_{i=\text{1}}^{N}{\left|b_{i}\right|}^{\text{2}}}{\sum_{i=\text{1}}^{N}{\left|a_{i}\right|}^{\text{2}}}} \end{array}$$ (10)

Where,

$a_{i}$ = incident wave at port i

$b_{i}$ = incident wave at port i

N = No. of antenna ports

From Fig 15(a), the simulated TARC values are –32 dB, –18 dB, –28 dB and –16 dB at the four resonant frequencies 24.5 GHz, 33.5, 38 and 44 GHz.

CCL is another metric, which measures the loss in channel capacity due to correlation between antenna patches in a MIMO system. A lower value indicates better diversity and higher system capacity. CCL [38] is given by


$$\begin{array}{c} CCL=-\sum_{i=\text{1}}^{N}{\text{log}}_{\text{2}}\left(\text{1}-{\left|\rho_{e,i}\right|}^{\text{2}}\right)\; \end{array}$$ (11)

Where,

$\rho_{e,i}$ = Envelope Correlation Coefficient (ECC) between antenna elements.

Fig. 15(b) presents the simulated CCL values are 0.02 bits/s/Hz, 0.12 bits/s/Hz, 0.05 bits/s/Hz, and 0.18 bits/s/Hz at 24.5, 33.5, 38, and 44 GHz, respectively, all of which are well below the acceptable threshold of 0.4 bits/s/Hz, confirming negligible channel capacity loss and excellent diversity performance of the modelled MIMO antenna.

Fig 16 illustrates the Mean Effective Gain (MEG) performance of the proposed MIMO antenna system, comparing both predicated and tested outcomes across the frequency range of 20–48 GHz. MEG [39] represents the sum of transmitted power of an antenna patch in a multipath environment, normalized to the incident power. It is a crucial diversity parameter employed to calculate the practical performance of MIMO systems. MEG is formulated as


$$\begin{array}{c} {MEG}_{i}=\frac{\text{1}}{\text{2}}\left(\text{1}-\sum_{j=\text{1},\;j=\;\;/\;i}^{N}{\left|S_{ij}\right|}^{\text{2}}\right),\;i=\text{1},\text{2},\ldots .\;N \end{array}$$ (12)

For the sake of high performance of the MIMO system, the MEG difference between antenna patches should be within 3 dB. The predicated Mean Effective Gain (MEG) magnitudes of the suggested MIMO antenna are approximately –3.2 dB, –4.0 dB, –4.5 dB, and –4.2 dB at 24.5, 33.5, 38, and 44 GHz, respectively. These magnitudes fall within the acceptable range of –3 dB to –5 dB, with only minimal variation among the antenna elements. Since the MEG difference is well below the 3 dB limit, the results confirm balanced power reception across all ports, ensuring efficient diversity performance and reliable operation of the MIMO antenna in multipath environments.

## 4 Conclusion

A 4-element compact MIMO antenna employing serrated boundary fractal geometry along with tuning fork slot. and a peripherally notched octagonal parasitic disc at the center has been designed and validated for quad-band operation at 24.5, 33.5, 38, and 44 GHz, targeting 5G and transitional 6G applications. The antenna achieves efficient impedance matching with return loss values below –10 dB and high isolation above 17 dB between elements, confirming effective suppression of mutual coupling. Diversity analysis further validates its performance: ECC values on the order of 10^-6^ result in a consistent diversity gain close to 10 dB, while TARC values remain below –10 dB, ensuring efficient multi-port excitation. In addition, CCL values are well below the 0.4 bits/s/Hz threshold, confirming negligible channel capacity loss, and MEG values within –3 to –5 dB with variations under 3 dB demonstrate balanced power reception across all elements. The antenna also exhibits peak gains of 8.45 dBi at 24.5 GHz, 10.01i dB at 33.5 GHz, 9.23 dBi at 38 GHz, and 10.47 dBi at 44 GHz, highlighting its capability to deliver strong radiation performance. Overall, the combination of serrated fractal boundaries tuning fork slot and the central notched octagonal disc enhance impedance bandwidth, gain, isolation, and diversity performance, making the suggested 4-element MIMO antenna a highly reliable candidate for next-generation high-capacity modern wireless communication networks.

## References

[1] Wang R, Yang Y, Makki B, Shamim A. A Wideband Reconfigurable Intelligent Surface for 5G Millimeter-Wave Applications. IEEE Trans Antennas Propagat. 2024;72(3):2399–410. doi: 10.1109/tap.2024.3352828

[2] Al-Gburi AJA. Coin-sized Dual-band Millimeter-Wave (mmWave) Antenna with Machine-learning-guided Impedance Prediction. PIER M. 2025;136:1–12. doi: 10.2528/pierm25071303

[3] Huang H-C, Lu J. Evolution of Innovative 5G Millimeter-Wave Antenna Designs Integrating Non-Millimeter-Wave Antenna Functions Based on Antenna-in-Package (AiP) Solution to Cellular Phones. IEEE Access. 2021;9:72516–23. doi: 10.1109/access.2021.3077309

[4] Al-Gburi AJA. Do We Really Need Frequency‐Selective Surface and Metasurface Reflectors for Antenna Gain Enhancement, or Are Metallic Reflectors Enough?. International Journal of Antennas and Propagation. 2026;2026(1). doi: 10.1155/ijap/2141943

[5] Sharma N, Singh HS, Khanna R. Design and Analysis of Multiband Fractal Antenna for MIMO/Diversity Applications. Wireless Pers Commun. 2021;122(4):3671–86. doi: 10.1007/s11277-021-09106-7

[6] Shet K, Karthikeya GS, Suraj HS. A wideband probe-fed low-cost mm wave fractal antenna array for 5G. Journal of Physics: Conference Series. 2020;1706:012094.

[7] Azzouz A, Bouhmidi R, Chetioui M, Berber R, Al-Gburi AJ. Performance optimization of crossed bowties of inverted sierpinski fractal antenna for multiband wireless communication applications. Telematics and Informatics Reports. 2025;:100231.

[8] Kumar A, Dewan B, Jain AK, Rawat P, Zakaria Z, Al-Gburi AJA. Design and development of four port wideband high isolation Koch curve fractal MIMO antenna. Progress In Electromagnetics Research B. 2025;112:15–27. doi: 10.2528/PIERB25040803

[9] Karmakar A. Fractal antennas and arrays: a review and recent developments. Int J Microw Wireless Technol. 2020;13(2):173–97. doi: 10.1017/s1759078720000963

[10] Malallah R, Shaaban RM, Al-Tumah WAG. A dual band star-shaped fractal slot antenna: Design and measurement. AEU - International Journal of Electronics and Communications. 2020;127:153473. doi: 10.1016/j.aeue.2020.153473

[11] Ghalamkari B, Mokhtari N. Wide-band octagon-star fractal microstrip patch antenna for terahertz applications. Optik. 2022;259:168990. doi: 10.1016/j.ijleo.2022.168990

[12] Kumar A, Singh AP. Design of micro‐machined modified Sierpinski gasket fractal antenna for satellite communications. Int J RF Microw Comput Aided Eng. 2019;29(8). doi: 10.1002/mmce.21786

[13] Bhutani P, Sagar S, Kumar A. Performance analysis of Sierpinski carpet fractal antenna for wireless communication. Appl Comput Automd Wirel Syst Electr Eng. 2019;553:749–58.

[14] Kaur M, Sivia JS. Minkowski, Giuseppe Peano and Koch curves based design of compact hybrid fractal antenna for biomedical applications using ANN and PSO. AEU - International Journal of Electronics and Communications. 2019;99:14–24. doi: 10.1016/j.aeue.2018.11.005

[15] Pérez-Moroyoqui R, Rodríguez-Romo S, Ibáñez-Orozco O. Patterns of the radiation properties for Peano antennas. Sci Rep. 2023;13(1):8358. doi: 10.1038/s41598-023-35185-6 37225759 PMC10209191

[16] Kubacki R, Czyżewski M, Laskowski D. Minkowski Island and Crossbar Fractal Microstrip Antennas for Broadband Applications. Applied Sciences. 2018;8(3):334. doi: 10.3390/app8030334

[17] Li-Na C, Yong-Chang J, Huan-Huan X, Fu-Shun Z. Minkowski fractal patch antenna for size and radar cross-section reduction. In: Proceedings of 2011 IEEE CIE International Conference on Radar, 2011. 1406–9. doi: 10.1109/cie-radar.2011.6159822

[18] Yu Z, Chang Y, Niu R, Zhang R, Wang F, Sun R, et al. A New Koch and Hexagonal Fractal Combined Circular Structure Antenna for 4G/5G/WLAN Applications. Electronics. 2025;14(2):237. doi: 10.3390/electronics14020237

[19] Paun M, Nichita M, Paun V, Paun V. Fifth‐generation fractal antenna design based on the Koch Snowflake geometry. A fractal theory application. Expert Systems. 2023;42(1). doi: 10.1111/exsy.13242

[20] Palandoken M, Gocen C. A modified Hilbert fractal resonator based rectenna design for GSM900 band RF energy harvesting applications. Int J RF Microw Comput Aided Eng. 2019;29(1):e21643. doi: 10.1002/mmce.21643

[21] Biswas B, Karmakar A, Chandra V. Hilbert curve inspired miniaturized MIMO antenna for wireless capsule endoscopy. AEU - International Journal of Electronics and Communications. 2021;137:153819. doi: 10.1016/j.aeue.2021.153819

[22] Ez-Zaki F, Belahrach H, Ghammaz A. Broadband microstrip antennas with Cantor set fractal slots for vehicular communications. Int J Microw Wireless Technol. 2020;13(3):295–308. doi: 10.1017/s1759078720000719

[23] Elabd RH, l-Gburi AJA. Design and Optimization of a Circular Ring-Shaped UWB Fractal Antenna for Wireless Multi-Band Applications Using Particle Swarm Optimization. Progress In Electromagnetics Research B. 2024;106:101–12. doi: 10.2528/PIERB24033002

[24] Behara SH, Gupta AK, Bammidi K, Edubilli SD, Gatteem A, Chowdary PSR. Sierpinski gasket fractal-based microwave metamaterial absorber. In: Visakhapatnam, India, 2024.

[25] Babu Kamili J, Addepalli T, Perli BR, Kiran Kumar B, Mohammed YT. Design of a novel four-element Koch–Sierpinski fractal mmWave antenna for 5G applications. International Journal of Electronics. 2023;111(12):2085–105. doi: 10.1080/00207217.2023.2248662

[26] Saleem I, Rafique U, Agarwal S, Savci HŞ, Abbas SM, Mukhopadhyay S. Ultra-Wideband Fractal Ring Antenna for Biomedical Applications. International Journal of Antennas and Propagation. 2023;2023:1–9. doi: 10.1155/2023/5515263

[27] Subramanian SN, Nataraj C, Duraikannan S, Selvaperumal SK, Abdulla R. Multiband Fractal Antenna for 26/28 GHz Millimeter Wave Band. In: 2022 International Conference on Edge Computing and Applications (ICECAA), Tamilnadu, India, 2022. 318–22.

[28] Mallat NK, Jafarieh A, Noorollahi H, Nouri M. A novel fractal arrow-shaped mmwave flexible antenna for iot and 5g communication systems. PIER Letters. 2022;107:9–17. doi: 10.2528/pierl22052405

[29] Rodrigues A, Schlosser E, Heckler M. A Detailed Design Procedure for Printed Log-Periodic Antennas with Koch Fractal Dipoles. JCIS. 2024;39(2024):13–21. doi: 10.14209/jcis.2024.2

[30] Al-Gburi AJA, Ibrahim IBM, Zakaria Z, Nazli NFBM. Wideband microstrip patch antenna for sub 6 GHz and 5G applications. Przegląd Elektrotechniczny. 2021;97(11):26.

[31] Attioui S, El Aaoud SE, Ibnyaich S, Zeroual A. Minkowski Fractal Antenna Design with Defected Ground Structure for 5G Millimeter Wave Applications. In: 2024 International Conference on Global Aeronautical Engineering and Satellite Technology (GAST), 2024. 1–4. doi: 10.1109/gast60528.2024.10520808

[32] Sneha K, Agarwal V. Design and Analysis of Compact Four‐Port Fractal‐Loaded MIMO Antenna for 5‐g Application. Int J Communication. 2025;38(13). doi: 10.1002/dac.70206

[33] Benkhadda O, Reha A, Saih M, Harrou M. A New Compact Cantor Set Fractal MIMO Antenna for 5G Millimeter-Wave Applications. In: 2024 International Microwave and Antenna Symposium (IMAS), 2024. 1–4. doi: 10.1109/imas61316.2024.10818140

[34] Udeze NP, Orakwue SI, Ehikhamenle MI. Design of a Tri-band Minkowski Fractal MIMO Antenna for FR2 5G Applications. J Eng Res Rep. 2024;26(9):190–205. doi: 10.9734/jerr/2024/v26i91272

[35] Naik Ketavath K. Quad-port wheel-shaped MIMO patch antenna system deployed at UWB application for 6G terahertz communications. Photonics and Nanostructures-Fundamentals and Applications. 2025;:101430.

[36] Perli BR, Addepalli T, Divya G, Kumar MK, Sharma M, Raju C, et al. Serpent-Configured Quad-Port MIMO Antenna with Dual-Band Operation and Defected Substrate-Ground Structure for Millimeter-Wave Systems. J Infrared Milli Terahz Waves. 2025;46(7). doi: 10.1007/s10762-025-01057-5

[37] Addepalli T. Effective area reduction & surface waves suppression of a novel four-element MIMO antenna exclusively designed for dual band 5G sub 6 GHz (N77/N78 & N79) applications. Wireless Networks. 2025:1463–79.

[38] Fritz‐Andrade E, Jardon‐Aguilar H, Tirado‐Mendez JA. The correct application of total active reflection coefficient to evaluate MIMO antenna systems and its generalization to N ports. Int J RF Microw Comput Aided Eng. 2019;30(4). doi: 10.1002/mmce.22113

[39] Sharma M, Perli BR, Matta L, Addepalli T, Sharma K, Sibai FN. Flexible four-port MIMO antenna for 5G NR-FR2 tri-band mmWave application with SAR analysis. Sci Rep. 2024;14(1):29100. doi: 10.1038/s41598-024-79859-1 39582070 PMC11586418

[40] Bisht N, Malik PK, Das S, Islam T, Asha S, Alathbah M. Design of a modified MIMO antenna based on tweaked spherical fractal geometry for 5G new radio (NR) band N258 (24.25–27.25 GHz) applications. Fractal and Fractional. 2023;7(10):718. doi: 10.3390/fractalfract7100718

[41] Enahoro S, Sunday E, Uko M, Alabi S, Elias F, Unnikrishnan R. A metamaterial-grounded ultra-wideband cross-fractal mimo antenna for K, Ka, and mmwave applications. 2024. 10.2139/ssrn.4691838

[42] Rahman MA, Al-Bawri SS, Abdulkawi WM, Islam MT. Miniaturized tri-band integrated microwave and millimeter-wave MIMO antenna loaded with metamaterial for 5G IoT applications. Results in Engineering. 2024;24:103130. doi: 10.1016/j.rineng.2024.103130

[43] Kumar A, Kumar A, Pharwaha APS. On the Development of Super-Wideband Sierpinski Triangular Fractal Antenna. Wireless Pers Commun. 2024;134(1):119–31. doi: 10.1007/s11277-024-10890-1

[44] Kolte JC, Bansal P, Kumar A. Design of Novel Tri-band Antenna for Wi-Fi, 5G Sub-6, band and WiMAX application Using Characteristic Mode Analysis. Scientia Iranica. 2025;0(0):0–0. doi: 10.24200/sci.2025.64037.8725

[45] Kumar A, Pharwaha APS. Development of a Modified Hilbert Curve Fractal Antenna for Multiband Applications. IETE Journal of Research. 2020;68(5):3597–606. doi: 10.1080/03772063.2020.1772126

